# Survival after resection of brain metastases with white light microscopy versus fluorescence-guidance: A matched cohort analysis of the Metastasys study data

**DOI:** 10.18632/oncotarget.27688

**Published:** 2020-08-11

**Authors:** Abdelhalim Hussein, Veit Rohde, Christina Wolfert, Silvia Hernandez-Duran, Ingo Fiss, Annalen Bleckmann, Alonso Barrantes Freer, Dorothee Mielke, Bawarjan Schatlo

**Affiliations:** ^1^Department of Neurosurgery, University of Medicine Goettingen, Goettingen, Germany; ^2^Department of Hematology-Oncology, University of Medicine Goettingen, Goettingen, Germany; ^3^Institute of Neuropathology, University of Medicine Goettingen, Goettingen, Germany; ^4^Department of Neuropathology, University Medical Center Leipzig, Leipzig, Germany; ^5^Department of Medicine A, University Hospital Muenster, Muenster, Germany; ^*^These authors share senior authorship equally

**Keywords:** brain tumor, brain metastasis, fluorescence-guided surgery, 5-ALA

## Abstract

Background: Metastatic brain disease continues to have a dismal prognosis. Previous studies achieved a reduction of local recurrence rates by aggressively resecting the peritumoral zone (supramarginal resection) or using 5-aminolaevulinic acid (5-ALA) fluorescence. The aim of the present study is to assess whether the use of 5-ALA has an impact on local recurrence or survival compared to conventional white light microscopic tumor resection.

Materials and Methods: We included consecutive patients who underwent surgical resection of brain metastases. Two groups were compared: In the “white light” group, resection was performed with conventional microscopy. In the 5-ALA group, fluorescence guided peritumoral resection was additionally performed after standard microscopic resection. In-brain recurrence and mortality were compared between groups.

Results: *N* = 175 patients were included in the study. All baseline parameters were similarly distributed with no significant difference between surgical groups. Local in-brain recurrence occurred in 21/175 patients (12%) with a rate of 15/119 (12.6%) in the white light and 6/56 (10.7%) in the 5-ALA group (*p* = 0.720). The use of 5-ALA influenced neither in-brain recurrence (OR 0.59 [CI = 95% 0.18; 1.99], *p* = 0.40) nor mortality (OR 0.71 [CI = 95% 0.27; 1.85], *p* = 0.49).

Conclusions: The use of 5-ALA did not result in lower in-brain recurrence or mortality compared to the use of white light microscopy. The most prominent predictors of survival remain favorable preoperative performance status, a low tumor diameter and the absence of multiple cerebral lesions.

## INTRODUCTION

Metastatic brain disease is more common than primary brain tumors [1]. Ten to 40% of cancer patients are eventually affected, equaling about 150,000 patients in Europe every year [2]. Cerebral dissemination is the most common cause of tumor-related death [3]. Radiotherapy and surgical lesion removal are the mainstay of treatment [4]. In a bid to improve local control, two surgical strategies have been advocated: One group suggested that en bloc-resection was superior to piecemeal [5]. Another group made the case for extending tumor resection 5 millimeters into peritumoral tissue to perform a so-called supramarginal resection [6].

5-aminolevulinic acid (5-ALA) is widely used to guide resection of high-grade gliomas. Its aim is to prolong progression-free survival through radical resection and improved local tumor control [7]. 5-ALA-uptake in cerebral metastases was first described by Utsuki et al. in 2007 [8] and recently confirmed histologically in the peritumoral zone by our group [9]. In a series of 52 patients, Kamp and colleagues detected positive fluorescence in 62% of resected cerebral metastases. Others, however, reported lower rates of positive intraoperative fluorescence [10]. Local recurrence rates were reportedly lower when surgeons were guided by positive fluorescence intraoperatively (5-ALA positive: 7% vs. 5-ALA negative: 23%). This observation failed to reach statistical significance and did not translate into better overall survival [11]. Thus, the utility and importance of using methods to improve local control of brain metastases remains an unresolved issue. The aim of the current study was to compare survival and local recurrence in a cohort of patients who underwent surgery for brain metastases with 5-ALA fluorescence microscopy to one that was operated using microscopic white light only.

## RESULTS

### Patient inclusion and exclusion

Out of 205 consecutive patients with brain metastases, 175 were included. Thirty cases (15%) were excluded: In 18 of the 30 cases (60%), the surgery was performed with the aim of obtaining a tumor biopsy and not of resecting the target lesion. In two cases (7%), the surgical procedure was not a tumor resection. Ten of the 30 patients (33%) were excluded because of in-hospital mortality less than 7 days after surgery.

### Patients excluded because of biopsy

The decision to perform biopsy was taken in eighteen patients: 12/18 because of eloquence and 6/18 because of tumor dissemination. The tumors in the biopsy group were located in the central region in *n* = 5, frontal lobe in *n* = 5, parietal lobe in *n* = 2, temporal lobe in *n* = 1, occipital lobe in *n* = 1, and cerebellum in *n* = 4 cases. Results of the biopsies showed small cell lung cancer in *n* = 2, melanoma in *n* = 2, breast cancer in *n* = 1, hepatic neoplasia in *n* = 1 case. The twelve remaining patients were initially included because of a prior medical history of cnacer. However, the pathological results failed to produce any evidence of a metastasis. Instead, we found brain hemorrhage (*n* = 1), abscess (*n* = 1), lymphoma (*n* = 1), subependymoma (*n* = 1) and glioblastoma (*n* = 8), prompting exclusion of these cases from the analysis. The five patients with central lesions received stereotactic procedure, and 13 underwent a navigated biopsy through a burrhole. In twelve patients (66.7%), the decision not to perform a biopsy was based on eloquent lesion localization. In six patients (33.3%), the number of cerebral lesions was too high to warrant surgical resection of a single target lesion.

### Patients excluded because of early death

Ten patients were enrolled in the study but were excluded in the present analysis because they did not survive the first week after surgery. 5-ALA was given in 2 of these ten patients (20%). Five of the early deaths (50%) were attributable to surgical complications. One patient had a massive brain edema because of cortical infarct in the middle cerebral artery territory. Four patients developed postoperative brain hemorrhage and died shortly thereafter. The other five patients (50%) died of medical causes: Sepsis (*n* = 1), internal hemorrhage (*n* = 2) and respiratory failure (*n* = 2).

### Baseline characteristics

Out of the 175 patients included in the study, 95 (54.3%) were male. Mean age was 63.5 years with a range of 35–85 years. Overall, 70.3% of patients (*n* = 123) had a preoperative KPS of ≥ 80. The resected lesion was in an eloquent region in 46 patients (26%). Tumor types were non small-cell lung cancer in 63 (36%), small cell lung cancer in 20 (11.4%), gastrointestinal neoplasms in 25 (14.3%), breast cancer in 26 (14.9%), melanoma in 25 (14.3%) and other neoplasms in 16 (9.1%) cases. Tumor diameter was ≥ 30 mm in 86 patients (49.4%). *N* = 46 patients had brain tumors located in an eloquent brain region (26.3%). Single brain lesions were recorded in 86 patients (49.1%), while the remainder of patients had multiple cerebral metastases. 5-ALA was applied in 56 patients (32%). All baseline parameters were similarly distributed with no significant difference between surgical groups (white light or 5-ALA; Table 1). Adjuvant therapies were balanced across surgical groups. Overall, 151 patients (86.3%) underwent additional radiotherapy (white light: 102/119 vs. 5-ALA: 49/56; *p* = 0.749) and 115 patients (65.7%) received chemotherapy. As in the baseline characteristics, an analysis of the adjuvant treatment regimen both surgical groups were subjected to did not reveal imbalances.

**Table 1:** Baseline characteristics

		White Light		5-ALA		Total		*p*-value
		*N*	(%)	*N*	(%)	*N*	(%)	
**Age**	**> 65 years**	52	44%	24	43%	76	43%	0.917
	**≤ 65 years**	67	56%	32	57%	99	57%	
**Sex**	**Female**	53	45%	27	48%	80	46%	0.649
	**Male**	66	55%	29	52%	95	54%	
**KPS**	**< 80**	37	31%	15	27%	52	30%	0.561
	**≥ 80**	82	69%	41	73%	123	70%	
**Histology**	**NSCLC**	40	33%	23	41%	63	37%	0.491
	**GIT**	18	15%	7	13%	25	14%	
	**Breast**	19	16%	7	13%	26	15%	
	**Melanoma**	14	12%	11	20%	25	14%	
	**SCLC**	16	13%	4	6.5%	20	11%	
	**Other**	12	11%	4	6.5%	16	9%	
**Disease status**	**Disseminated**	65	55%	23	41%	88	50%	0.094
	**Not dissem.**	54	45%	33	59%	87	50%	
**Eloquence**	**Non-eloquent**	89	75%	40	71%	129	74%	0.637
	**Eloquent**	30	25%	16	29%	46	26%	
**Diameter**	**> 30 mm**	61	51%	25	45%	86	49%	0.476
	**≤ 30 mm**	58	49%	30	55%	88	51%	
**Brain lesions**	**Multiple**	63	53%	26	46%	89	51%	0.421
	**Single**	56	47%	30	54%	86	49%	
**Location**	**Supratentorial**	66	56%	42	75%	108	62%	0.013
	**Infratentorial**	53	45%	14	25%	67	38%	
**Total**		**119**		**56**		**175**		

### Intraoperative fluorescence in the 5-ALA group

**Figure 1:**
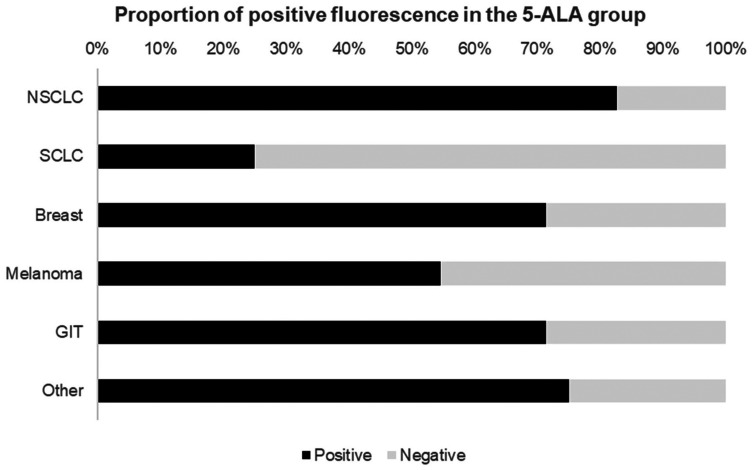
Proportion of positive fluorescence in patients who received 5-ALA prior to induction of surgery showed intraoperative fluorescence in approximately 69% of cases. This bar graph depicts the relative proportions of positive fluorescence by primary tumor/histology. Abbreviations: NSCLC: Non-small cell lung cancer; SCLC: Small-cell lung cancer, GIT: Gastrointestinal tumor.

Tumor fluorescence was found to be present in 39 out of 56 patients who were operated in the 5-ALA group (69.6%), while the remaining patients (17/56; 30.4%) showed no sign of fluorescence intraoperatively. Whether a tumor was fluorescent during surgery differed depending on primary tumor type, although there was no significant association with a specific tumor (*p* = 0.231). Positive fluorescence was present in one out of four small cell lung cancer cases (25%), in 19/23 non-small cell lung cancers (83%), in 5/7 gastrointestinal tumors (71%), in 5/7 breast cancers (71%), in 6/11 melanomas (55%), and in 3/4 of the remaining cases (75%; Figure 1).

### Overall and local in-brain recurrence

Mean follow up was 54.8 ± 31.2 weeks. During the follow up period, 135 (77.1%) patients died. Mean survival was 44.6 ± 45 weeks. The white light-group had a mean survival of 45.5 ± 48 weeks. Survival in the 5-ALA amounted to 43.6 ± 40 weeks (*p* > 0.05). The overall long-term survival rate (> 24 months) was 9.1% (*n* = 16). In the white light group, 12 patients out of 119 were long term survivors (10%), while 4 out of 56 (7%) were long-term survivors in the 5-ALA group (*p* = 0.529).

**Figure 2:**
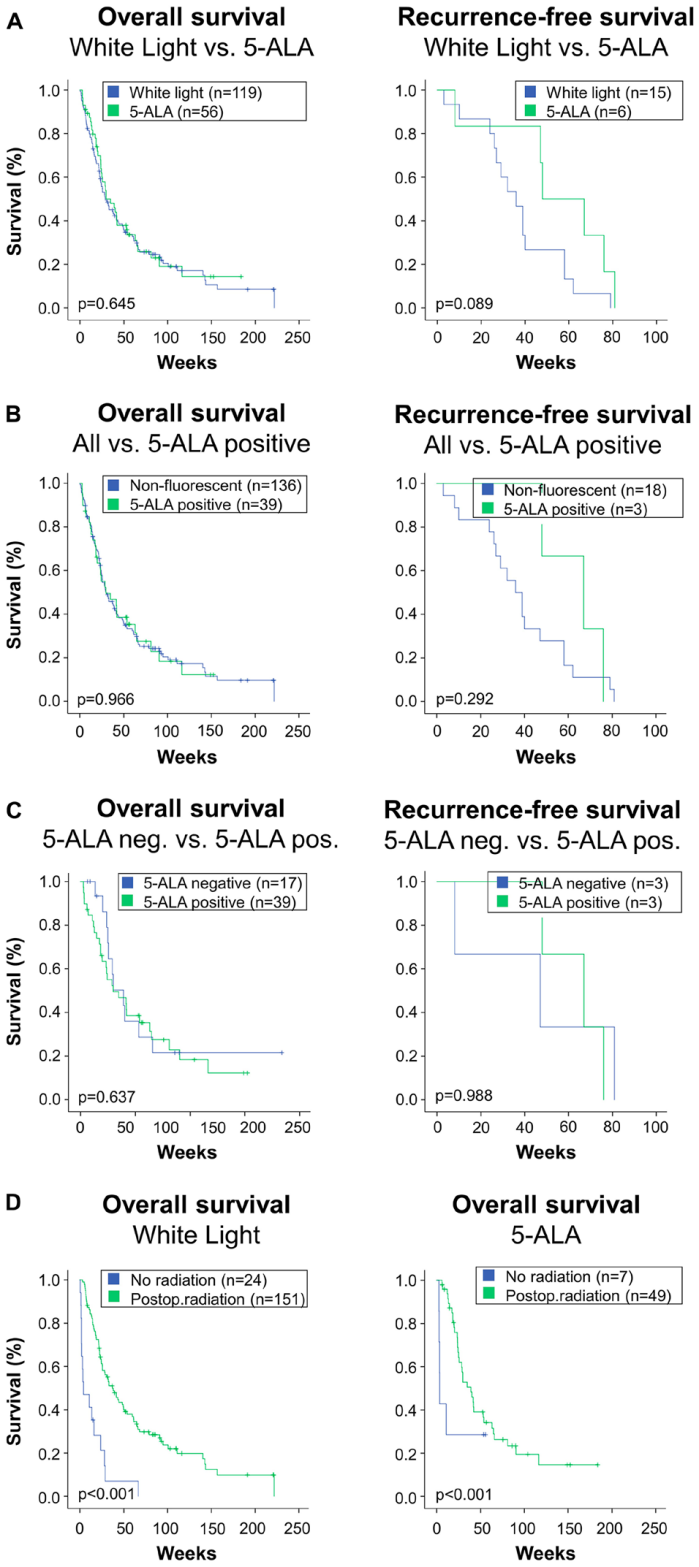
This figure depicts overall survival and local in-brain recurrence-free survival in the study’s subgroups. (**A**) Patients who were operated under microscopic white light were compared to patients who received 5-ALA. The 5-ALA group comprises all patients receiving the fluorescent dye, irrespective of the actual intraoperative finding of fluorescence. Log rank comparison showed no difference in overall survival (left), while there was a trend towards higher recurrence-free survival in the 5-ALA group (right). (**B**) About one third of patients who received 5-ALA did not exhibit fluorescence during surgery. Therefore, although they were attributed to the 5-ALA group in the analysis, the surgeon was not able to use fluorescence. Therefore, an “as treated” analysis was performed. Therein, all patients who were 5-ALA negative, i. e. received 5-ALA but showed no fluorescence intraoperatively, were added to the White Light-group. This results in a 5-ALA positive versus “all others” or “non-fluorescent” comparison. This analysis revealed no difference in overall or recurrence-free survival between groups in the Kaplan log rank comparisons. (**C**) For a subgroup analysis, patients who received 5-ALA were split in two groups. The first one represents the lack of intraoperative fluorescence (5-ALA neg.), and the other includes patients where red fluorescence was visible (5-ALA pos.). This subgroup analysis revealed no inter-group differences in overall or recurrence-free survival. (**D**) Radiotherapy significantly impacts survival. For internal data validation purposes, the impact of radiotherapy was compared in the study’s main groups: White light microscopy (left) versus patients who received 5-ALA (right). In both groups, the marked effect of radiotherapy was reconfirmed.

Local in-brain recurrence occurred in 21/175 patients (12%) with a rate of 15/119 (12.6%) in the white light and 6/56 (10.7%) in the 5-ALA group (*p* = 0.720). Recurrence-free survival overall was 42.3 ± 23 weeks, while it was 37.5 ± 20 weeks in the white light and 54.5 ± 27 weeks in the 5-ALA group (*p* > 0.05). Kaplan Meier log rank comparison revealed no inter-group differences in overall or progression-free survival with a visible trend towards delayed in-brain progression in the 5-ALA group (Figure 2, *p* = 0.089). This trend, however, abated after multivariate correction, where both in-brain recurrence (OR 0.59 [CI = 95% 0.18; 1.99], *p* = 0.40) and mortality (OR 0.71 [CI = 95% 0.27; 1.85], *p* = 0.49) had no tangible association with resection technique.

### Radiotherapy subgroup and as-treated analysis

In order to compensate for the potential role of adjuvant radiotherapy [11], we conducted a subgroup analysis on patients who underwent additional radiotherapy. Local in-brain tumor recurrence showed a favorable trend for the 5-ALA group (Figure 2, *p* = 0.089). The identical graphs in the with- and without radiotherapy groups is explained by the lack of local in-brain recurrence in the 5-ALA no-radiotherapy-group.

Just over two thirds of patients with 5-ALA displayed positive tumor fluorescence during surgery. Therefore, the comparison of 5-ALA versus white light groups may effectively portray an “intent-to-treat” analysis. The cases where fluorescence failed to appear were operated with white light only. Consequently, for the sake of a post-hoc analysis, 5-ALA negative cases were transferred to the white light group. One may argue that an “as-treated” analysis representing positive fluorescence is helpful to understand a potential effect of 5-ALA-guidance. Thus, we conducted a post-hoc analysis dichotomizing the patient population into “white light plus 5-ALA-negative” (*n* = 136) and “5-ALA positive” (*n* = 39). The results of this analysis, shown in Figure 2, showed no differences in survival (*p* = 0.966) or local in-brain recurrence (0.292) in either group.

### Predictors of survival and recurrence-free survival

Since 5-ALA revealed no association with survival or in-brain recurrence, we searched for outcome predictors among our set of baseline characteristics (Table 2). Favorable parameters against case fatality were KPS > 70 (OR 0.19 [CI = 95% 0.06–0.63], *p* = 0.007), tumor diameter < 30 mm (OR 0.32 [CI = 95% 0.12; 0.81], *p* = 0.017) and the presence of not more than one brain lesion (OR 0.12 [CI = 95% 0.04–0.33], *p* < 0.001). Comparable predictors of in-brain recurrence, however, were not identified.

**Table 2:** Predictors of case fatality and recurrence

		Risk of death				Risk of local in-brain recurrence			
		OR	CI (5–95%)		*p*-value	OR	CI (5–95%)		*p*-value
**Baseline**	**Age ≤ 65 years**	0.69	0.28	1.38	0.435	3.40	1.02	11.37	0.047
	**Male sex**	1.76	0.63	4.93	0.280	1.06	0.31	3.70	0.924
	**KPS > 70**	0.19	0.06	0.63	0.007	1.49	0.42	5.35	0.540
**Histology**	**NSCLC**	Ref	Ref	Ref	0.014	Ref	Ref	Ref	0.082
	**GIT**	2.28	0.39	13.14	0.358	6.73	0.56	81.60	0.134
	**Breast**	0.48	0.12	1.94	0.300	16.19	1.48	177.74	0.023
	**Melanoma**	0.31	0.08	1.21	0.092	29.63	3.11	282.41	0.003
	**SCLC**	7.27	0.76	69.82	0.086	11.73	1.05	131.09	0.046
	**Other**	0.12	0.02	0.55	0.007	8.98	0.72	112.65	0.89
**Systemic**	**Not Disseminated**	0.96	0.39	2.39	0.930	1.36	0.48	3.86	0.568
**Radiological**	**Eloquent**	0.97	0.36	2.67	0.959	0.70	0.21	2.34	0.565
	**Diameter ≤ 30 mm**	0.32	0.12	0.81	0.017	1.19	0.42	3.35	0.742
	**Single brain lesion**	0.12	0.04	0.33	< 0.001	1.19	0.42	3.43	0.740
	**Infratentorial**	0.38	0.14	1.04	0.061	0.70	0.21	2.34	0.565
**Surgical**	**5-ALA**	0.71	0.27	1.85	0.485	0.59	0.18	1.99	0.400

## DISCUSSION

The present analysis was conducted to assess the impact of fluorescence-guided resection on overall survival in patients with brain metastases in a large series. In a disease fraught with systemic confounders, we found no association of the use of fluorescence and overall survival or in-brain recurrence. Preoperative KPS, solitary brain lesion and small tumor size were the most potent predictors favouring survival.

### Response rates to 5-ALA

In our study, the overall fluorescence rate reached 69%. Each tumor type had a distinct response rate to 5-ALA with fluorescence rates from 25% (small cell lung cancer) to 83% (non-small cell lung cancer). An unresolved issue is that reported rates of fluorescence in metastases vary strongly. In the recently published series from Kamp and colleagues only about 28% of a cohort of 218 patients showed positive intraoperative fluorescence [12]. In the aforementioned series, half of the patients suffered from non small-cell lung cancer. About 8–12% had melanoma, gastrointestinal and breast malignancies respectively. In our series, non small-cell lung cancer was the primary in just over one third of patients, while the remaining tumors showed similar distributions. Since non small-cell lung cancer showed the highest rate of fluorescence in our study, we cannot conclude that a higher rate of tumors with higher fluorescence was the cause of the discrepancy in fluorescence rates.

When comparing the group that was fluorescence-positive to the negative group, they found a difference in survival and recurrence rate favouring the fluorescence positive group. Besides the negligibly altered time interval of 5-ALA administration which was three hours prior to surgery in the series of Kamp et al. compared to four hours in ours, this difference cannot be explained. Meta-analyses or prospective studies focusing on specific types of metastases with standardized protocols including a reproducible cut-off for positive fluorescence [13] may improve our understanding of this heterogeneity.

### Of gliomas and metastases

The use and utility of 5-ALA in glioma surgery is well established. Despite a large infiltration zone that has most likely eluded surgery at any given timepoint of resection, gliomas remain localized to the brain. Nine out of ten gliomas recur locally [14], corroborating the need for local control. In contrast, metastases are a systemic disease in which local control in the brain may on occasion play second fiddle. Brain metastases have a low rate of local in-brain recurrence, affecting one in eight patients in our series. Depending on the viewing angle, this can be interpreted as a sign of surgical success. Alternatively, the patient may not have survived to witness local in-brain recurrence. It remains difficult to extract meaningful organ-specific progression-free survival data from aggregate endpoints such as systemic disease progression [15].

### The role of refined microsurgery in the treatment of brain metastases

Supra-marginal resection enables significantly improved local tumor control compared to standard gross total resection [5] without increasing complication rates [16]. The trend towards better local in-brain recurrence in the 5-ALA group may support this conception. On the other hand, it becomes increasingly clear that while better local control is achievable through refinement of resection techniques, improvements in overall survival are unlikely to be spearheaded by surgical intervention alone.

Individually tailoring treatment for each patient is crucial. A better performance score remains the strongest predictor of a more favourable disease course. In the comment on an article on patient selection, commentators came close to writing off the role of microsurgery in the treatment of cerebral metastases as a whole [16]. Over a decade later, we continue to operate regularly on brain metastases. In specific cases, such as melanomas with BRAF mutations, the need for adjuvant radiotherapy may even be put into question. Nonetheless, surgical outcomes are dependent on radiotherapy. Therefore, a separate subgroup analysis was performed in this study as suggested by others to dissect out the impact of radiation in the white light and 5-ALA groups [11]. The present study confirmed that regardless of surgical adjunct, radiotherapy is strongly associated with improved survival.

### Limitations

The interpretation of outcome data from studies on metastatic brain disease requires caution. As in our series, many datasets mix lung, skin, breast and intestinal neoplasms. Each of the aforementioned entities are entitled to their own molecular and clinical subcategorizations. Their pooling for the sake of producing brain-specific endpoints is justified by the clinicians’ need to understand how patients fare after treatment.

## MATERIALS AND METHODS

### Inclusion and exclusion criteria

This is a retrospective analysis based on the Metastasys study data. We included consecutive patients suffering from metastatic brain disease who underwent surgery. Patients were prospectively enrolled in the Metastasys study between June 2013 and December 2016 in accordance with the Ethics committee of the Georg-August University, Goettingen, approval no. 24/10/05. Patients were excluded from the present analysis if 1) they were younger than 18 years of age, 2) patients were unable or unwilling to consent by themselves or via an authorized legal representative, 3) if the goal of surgery was not the resection of the target lesion, but e.g. simply obtaining histopathological specimen through a biopsy, 4) if follow-up in surviving patients was < 1 year and 5) if patients died within the first week of surgery, thus obviating a meaningful survival analysis.

### Baseline characteristics

Sex, age and Karnofsky performance status (KPS) were recorded. KPS was dichotomized with KPS of ≥ 80 defined as good and a KPS < 80 as poor, respectively. Location and size of the tumor were included in the analysis. Tumor size was dichotomized using maximal axial lesion diameter: large (≥ 30 mm) and small (< 30 mm). Location of the resected lesion was dichotomized into eloquent and non-eloquent brain region. The following regions were considered eloquent: precentral or postcentral gyrus, calcarine fissure, left frontal operculum, left inferior parietal lobule, left superior temporal gyrus (posterior part), dentate gyrus, internal capsule, basal ganglia, thalamus, and hypothalamus [17]. Radiation therapy, extracerebral metastases and the number of cerebral metastases were recorded as well.

### Surgical groups and intraoperative use of 5-ALA

The decision to perform surgery was made based on guidelines and an interdisciplinary tumor board [18]. Neuronavigation was used to guide the resection in all cases. If necessary, intraoperative electrophysiological neuromonitoring was used at the discretion of the surgeon. Two groups were compared. In the first group, tumor resection was performed conventionally using microsurgical technique, i.e. “white light”. In the second group, patients were operated using 5-ALA. The availability of a 5-ALA microscope determined whether a patient was operated on using fluorescence. We routinely used 5-ALA in the resection of brain metastases. However, when two tumor surgeries per day were performed, glioma cases were given priority over metastases. Therefore, logistic reasons led to the fact that some patients underwent 5-ALA guided resection (5-ALA group) while others underwent microsurgical resection without the use of fluorescence (white light group). 5-ALA was administered orally four hours prior to intubation with a dose of 20 mg/kg as described elsewhere [19]. In the 5-ALA group, fluorescence guidance was used once the surgeon deemed microsurgical tumor resection complete.

### Overall and progression-free survival

Survival was recorded in weeks and evaluated at regular neuro-oncological follow-up visits. Patients were contacted by telephone in case of unavailable follow-ups. The communal population office was consulted prior to freezing the dataset to ensure that no patient deceased in the meantime. Time to death and in-brain recurrence were measured starting at the time of surgery. According to RANO criteria, the definition of in-brain recurrence was radiological evidence of tumor on follow-up imaging in or around the resection cavity [20]. Owing to our focus on surgical results in one organ, the calculation of PFS is problematic [15] and was therefore based solely on the occurrence of local in-brain recurrence.

### Statistical methods

The variable of interest was the surgical use of white light versus 5-ALA. Group imbalances were analyzed using two-tailed chi square tests. Local recurrence and survival analyses were performed using the a log rank test with a significance level of *p* ≤ 0.05. Multivariate binary logistic regression was used to identify predictors of survival. SPSS 23.0 (IBM Corp., 2015) was used for statistical analysis.

## CONCLUSIONS

In this large series on consecutive cases of surgically treated brain metastases, the use of 5-ALA did not lower in-brain recurrence compared to the use of white light microscopy. Overall survival was affected by other variables than surgical technique. The fine nuances of surgical technique which we attempt to study may have a comparatively low effect size. This is especially true in disseminated cancer in which non-brain-specific events may dramatically alter outcome. The most prominent predictors of survival remain favorable preoperative performance status, a low tumor diameter and the absence of multiple cerebral lesions. Developing a reproducible criterion on 5-ALA positivity may help resolve the current heterogeneity in clinical research on fluorescence-guided brain surgery.
